# Age-related differences in neural recruitment during the use of cognitive reappraisal and selective attention as emotion regulation strategies

**DOI:** 10.3389/fpsyg.2014.00296

**Published:** 2014-04-10

**Authors:** Eric S. Allard, Elizabeth A. Kensinger

**Affiliations:** Cognitive and Affective Neuroscience Laboratory, Department of Psychology, Boston CollegeChestnut Hill, MA, USA

**Keywords:** aging, emotion regulation, fMRI, selective attention, reappraisal

## Abstract

The present study examined age differences in the timing and neural recruitment within lateral and medial PFC while younger and older adults hedonically regulated their responses to unpleasant film clips. When analyses focused on activity during the emotional peak of the film clip (the most emotionally salient portion of the film), several age differences emerged. When comparing regulation to passive viewing (combined effects of selective attention and reappraisal) younger adults showed greater regulation related activity in lateral PFC (DLPFC, VLPFC, OFC) and medial PFC (ACC) while older adults showed greater activation within a region DLPFC. When assessing distinct effects of the regulation conditions, an ANOVA revealed a significant Age × Regulation Condition interaction within bilateral DLPFC and ACC; older adults but not young adults showed greater recruitment within these regions for reappraisal than selective attention. When examining activity at the onset of the film clip and at its emotional peak, the timing of reappraisal-related activity within VLPFC differed between age groups: younger adults showed greater activity at film onset while older adults showed heightened activity during the peak. Our results suggest that older adults rely more heavily on PFC recruitment when engaging cognitively demanding reappraisal strategies while PFC-mediated regulation might not be as task-specific for younger adults. Older adults' greater reliance on cognitive control processing during emotion regulation may also be reflected in the time needed to implement these strategies.

## Introduction

Despite normative declines in several areas of functioning (e.g., cognition and health) older adults report relatively high levels of emotional well-being (Carstensen and Mikels, 2005). One prevailing theory in the psychology of human aging suggests that older adults are motivated to maximize emotional well-being due to limits in future time perspective (Carstensen et al., 1999). Self-report studies have shown that older adults are more motivated to regulate emotion as compared to their younger counterparts (Kennedy et al., 2004; Kliegel et al., 2007). Furthermore, this enhanced motivation can be shown in reported attempts to actively enhance well-being through the use of particular emotion regulation strategies (Gross et al., 1997).

One of the most studied regulatory strategies is cognitive reappraisal. Cognitive reappraisal is a strategy that involves the reinterpretation of a stimulus/situation in order to change its meaning and emotional impact. Reappraisal draws upon processes associated with cognitive control and executive functioning (Ochsner and Gross, 2005). Several neuroimaging studies have identified a series of brain systems involved in active attempts to reappraise affect including dorsolateral, ventrolateral, and dorsomedial prefrontal cortex (PFC) and posterior parietal cortex (Ochsner et al., 2004; Kalisch, 2009; Winecoff et al., 2011; Silvers et al., 2013). These aforementioned regions have been shown to correspond with specific cognitive control processes likely to support emotion regulation (Ochsner and Gross, 2008). In a recent meta-analysis, Buhle et al. (2013) observed activity in regions of dorsolateral (DLPFC), ventrolateral (VLPFC), and dorsomedial (DMPFC) prefrontal cortex during reappraisal tasks. Their results supported predictions that DLPFC activation may support the manipulation of affective appraisals in working memory, VLPFC may aid the selection and inhibition of appraisals (e.g., selecting a non-affective appraisal or positive reappraisal while trying to inhibit a negative appraisal), and DMPFC may reflect processes related to updating the success of chosen appraisals (e.g., was the desired affective state obtained?). Older age is associated with declines in volumetric gray matter in several PFC regions (Raz et al., 2004; Grieve et al., 2005; Fjell et al., 2009), raising questions regarding whether older adults are able to use reappraisal strategies as successfully as younger adults (see Gross et al., 1997; John and Gross, 2004; Urry and Gross, 2010 for this debate).

Very few studies have directly assessed age differences in neural recruitment when performing specific emotion regulation strategies. Current evidence suggests that older adults activate PFC regions to a lesser extent than younger adults during hedonic regulation (e.g., diminishing negative affect) and that this limited activation might relate to older adults' reduced success at deploying reappraisal-type strategies. For instance, Opitz et al. (2012) had a group of younger and older adults perform hedonic and non-hedonic reappraisal (decrease and increase emotional reactions) in response to negative images in a series of fMRI tasks. In this study, older adults showed reduced DMPFC and VLPFC activation when reappraising negative images as compared to younger adults. Furthermore, diminished activation in VLPFC among older adults was accompanied by difficulty in decreasing negative responses to those images. Similar results were observed in another recent study in which older adults reported more negative affect in response to negative images when attempting to downreglate negative reactions as compared to younger adults (Winecoff et al., 2011). Furthermore, in this study, older adults showed reduced activation in a region of VLPFC when reappraising negative images as compared to younger adults. Taken together, these results suggest that older adults may be less effective than their younger adult counterparts in using reappraisal strategies to decrease negative affect (see also Tucker et al., 2012), perhaps because reappraisal relies on cognitive processes within lateral PFC that diminish with age (e.g., diminished VLPFC recruitment corresponding to a difficulty with inhibiting prepotent negative responses; Silvers et al., 2013).

Although past research has examined the effect of aging on neural mechanisms supporting cognitive reappraisal, less work has assessed the effects of aging on other antecedent strategies that may be less cognitively demanding. In their SOC-ER (selective optimization with compensation in emotion regulation) model, Urry and Gross (2010) proposed that older adults use less cognitively demanding regulatory strategies, such as situation selection/modification or attention deployment, rather than strategies such as reappraisal or suppression that rely heavily on cognitive control processing. This model fits well with evidence that older adults show a “positivity effect” (either positive engagement or negative avoidance) in visual attention in response to emotional stimuli (Isaacowitz et al., 2006), perhaps reflecting older adults' attempts to use selective attention as a regulatory strategy (Isaacowitz et al., 2008, 2009b) because it does not heavily impinge on cognitive resources (Allard et al., 2010). In cases where cognitive resources are constrained (e.g., in divided attention tasks), there is research suggesting that older adults will not/cannot preferentially engage with information in line with presumed emotion regulation goals (see Mather and Knight, 2005; Knight et al., 2007). Thus, emotion regulation processes might proceed more efficiently for older adults in situations where a full complement of cognitive control resources is available.

For the current study, we compared younger and older adults' neural recruitment while implementing two different types of antecedent emotion regulation strategies. We asked participants to regulate their emotions using selective attention or cognitive reappraisal. Given past research demonstrating older adults' diminished recruitment of lateral and medial PFC regions for certain forms of reappraisal as compared to younger adults, and their difficulty in using reappraisal to successfully regulate in real time (Opitz et al., 2012), we expected older adults in our sample to display reductions in the speed or level of recruitment of lateral and medial PFC during emotion regulation. However, it was less clear how the type of emotion regulation strategy would affect younger and older adults' recruitment of PFC regions. We used relatively long (40 s) film clips so we could examine a more protracted time course of emotion regulation. By using stimuli with a long duration, we could adjudicate between three plausible alternative outcomes regarding age-related changes in PFC recruitment. First, there may be a main effect of age, such that regardless of the strategy used, older adults may chronically under-recruit PFC when regulating their emotions, even when given time to implement the strategy. Second, there may be an interaction between age and time course for strategy implementation, such that older adults may require more time to recruit PFC processes, but when given sufficient time, they may recruit them in the same manner as younger adults. This prediction is based on previous evidence suggesting that the implementation of processes assumed to be in the service of emotion regulation (e.g., cognitive disengagement from negative stimuli) might take some time to execute for older adults (Isaacowitz et al., 2009a). Third, older adults' implementation of PFC processes may be affected by the task demands (i.e., type of regulatory strategy), yielding different effects of age and time course for cognitive reappraisal and selective attention.

To examine these alternatives, we recruited a group of younger (18–34) and older (55–85) adults to complete selective attention and cognitive reappraisal tasks during an fMRI scan session. We presented participants with a series of positive, negative, and neutral film clips within three scanning conditions: passive viewing, selective attention, and reappraisal. Emotional film clips were used in order to provide a dynamic, emotionally evocative stimulus set for examining age differences in neural recruitment in response to specific emotion regulation strategies. We focus our main analyses on the downregulation of negative affect in response to negative videos, consistent with the focus of the majority of studies that have compared younger (see meta-analysis by Diekhof et al., 2011) and older adults' neural responsivity to emotional inputs (particularly in the context of reappraisal: Urry et al., 2006, 2009; van Reekum et al., 2007; Opitz et al., 2012).

## Materials and methods

### Participants

Forty-five younger (25 female, *range* = 18–35 years; *M* = 23.40, *SD* = 4.39) and 42 older adults (25 females, *range* = 55–85; *M* = 69.21, *SD* = 8.62) participated in this study. Eleven younger adults and 12 older adults were excluded from subsequent analyses due to poor data quality (i.e., excessive motion artifacts: greater than ±5 mm of head motion; <20 motion outliers within each scan run as determined by an artifact correction procedure) or a failure to complete all three functional scans. The final sample included 34 younger (16 females, *M* = 23.79, *SD* = 4.33) and 30 older adults (20 females, *M* = 68.47, *SD* = 8.14) who had no history of psychiatric, neurological, or learning disorders nor any history or current use of psychiatric medication. Younger and older adults performed somewhat similarly on a variety of cognitive ability measures (younger adults outperformed older adults on only 2 out of 5 tasks assessing frontal lobe functioning derived from Glisky et al. (1995); see Table 1). Informed consent was obtained from all participants in accordance with the Boston College Institutional Review Board. All participants received $25/h. for their participation.

**Table 1:** **Additional demographic and cognitive variables**.

**Variable/Test**	**Young adults**		**Older adults**		***F***
	***M***	***SD***	***M***	***SD***	
Education	16.27	1.96	16.37	2.44	0.031
Shipley	33.54	3.01	35.37	3.17	5.07^*^
Digit back^a^	8.39	2.36	7.87	2.33	0.729
FAS^b^	48.07	10.82	48.03	12.59	0.00
WISC^c^	5.89	0.42	5.23	1.43	5.51^*^
Arithmetic^d^	15.68	3.39	15.03	2.75	0.638
Mental control^e^	30.69	4.15	26.10	5.49	7.24^*^

a-e Glisky frontal lobe tasks (Glisky et al., 1995).

* p < 0.05.

### Stimuli

We used a series of emotional and neutral film clips as stimuli for the fMRI scan sessions. We focused our analysis for the present study on the neural activity to the negative stimuli. The clips were obtained from television programs, feature films, and documentaries. Positive and negative clips included a variety of emotional scenarios. For instance, positive videos included amusing situations (e.g., standup comedy routine) or more tender/heartwarming scenarios (e.g., a married couple talking about how they first met). Negative videos included fear/sad/disgust scenarios (e.g., a woman being threatened on the phone; a man comforting his dying dog; a man digging through a messy toilet). A separate group of 14 younger and 14 older adults rated each clip on dimensions of valence and arousal. Ratings were made on a scale from 1 (*highly unpleasant, non-arousing*) to 9 (*highly pleasant, highly arousing*). Based on these ratings, a total of 45 clips (18 positive, 18 negative, 9 neutral) were selected for inclusion in the study. Positive and negative videos were matched on ratings of valence and arousal between younger and older adults: Positive-Valence (*M*_young_ = 7.31, *SD* = 0.63; *M*_old_ = 7.46, *SD* = 0.81; *p* = 0.45); Positive-Arousal (*M*_young_ = 6.19, *SD* = 0.46; *M*_old_ = 6.60, *SD* = 0.82; *p* = 0.10); Negative-Valence (*M*_young_ = 2.19, *SD* = 0.79; *M*_old_ = 2.12, *SD* = 1.14; *p* = 0.76); Negative-Arousal (*M*_young_ = 7.05, *SD* = 0.79; *M*_old_ = 7.19, *SD* = 0.69; *p* = 0.46); Neutral-Valence (*M*_young_ = 5.28, *SD* = 0.26; *M*_old_ = 5.47, *SD* = 0.39; *p* = 0.20). For each scan session, the order in which a particular clip was presented (whether it appeared in the passive viewing, selective attention, or reappraisal condition) was randomized across participants. Presentation of the clips was also pseudorandomized within each condition with the caveat that no more than three clips of the same valence were presented consecutively. Stimulus presentation was accomplished using SR Research EyeLink 1000 software (Kanata, Ontario, CA) during the scan session; although this presentation program acquired eye tracking data from participants, the eye tracking data are not reported here.

### Procedure

Before entering the MRI scanner, participants were instructed that they would view a series of emotional and neutral film clips during three functional scan runs. Within each run, six positive, six negative, and three neutral clips were presented. Participants were told that they would be given specific instructions on how to view the film clips with a series of instructions presented to them while in the scanner. Each regulation task (passive viewing, selective attention, and reappraisal) was performed within separate scan runs. For the “passive viewing” task, participants were instructed that they would view a series of 15 film clips; they should “view the clips naturally, as if at home watching television.” For the selective attention condition, participants were instructed to “focus on areas of the screen that would help increase positive and decrease any negatives feelings/reactions in response to the clips.” For the reappraisal condition, participants were provided with hedonic regulation instructions. When presented with negative clips, participants were instructed to utilize their choice of two strategies: detached reappraisal (“Try to distance yourself from the events being portrayed by reminding yourself that what you are viewing is a fictional event; these are just actors portraying a role.”) and positive reappraisal (“Try to put a positive spin on the outcome of the event being portrayed. For example, if you see a clip of a car accident, try to imagine that no one was seriously injured/killed, and everyone walked away from the accident relatively unharmed.”). We provided these strategies as options for participants given that certain clips might lend themselves to be more easily reinterpreted with one strategy or the other (or perhaps even both).

### Trial structure

A black fixation cross was shown on the center of a gray screen for 10, 12, 14, 16, or 18 s. Each video was presented for 40 s^1^. During the passive viewing condition, the instruction “view” was presented on the bottom of the screen 4-s post-stimulus onset and remained on the screen for 3 s. This timing for the instruction phase has been used in previous research (see Opitz et al., 2012). Using the same timing and screen placement, for the selective attention condition, the instruction “avoid negative” was presented along with the negative videos and for the reappraisal condition, “decrease negative” was presented. In all three conditions, each video was followed by an inter-trial interval consisting of a black fixation cross for an average of 14 s (jittered between 10 and 18 s). The presentation order of the three conditions was varied across participants.

### Data acquisitionand analysis

Images were acquired on a 3 Tesla Siemens Tim Trio MRI scanner using a 12-channel head coil. Stimuli were projected onto a screen located at the back of the magnet bore, and participants viewed stimuli using a mirror attached to the head coil. T1-weighted localizer images and a T1-weighted inversion recovery echo planar image required for auto-alignment were collected. Anatomical data were collected with a multiplanar rapidly acquired gradient-echo (MEMPRAGE) sequence (*TR* = 2200 ms; *TE*s = 1.64, 3.5, 5.36, 7.22; flip angle = 7°; FOV = 256 × 256 mm; slice thickness = 1 mm, no gap; 1 × 1 × 1 mm resolution). Functional images were collected using a T2^*^-weighted echo-planar imaging (EPI) sequence with the following parameters: *TR* = 2000 ms, *TE* = 30 ms, FOV = 216 mm, flip angle = 85°. Thirty interleaved near axial slices were collected in a 3 × 3 × 3.6 mm matrix (slice thickness = 3 mm with a 20% skip).

Preprocessing and data analysis were conducted in SPM8 (Welcome Department of Cognitive Neurology, London). Preprocessing steps were as follows: slice timing correction; motion correction using a six parameter, rigid body transformation algorithm; normalization to the Montreal Neurological Institute (MNI) template (resampling at 2 mm isotropic voxels); and spatial smoothing using a 8 mm full-width half maximum isotropic Gaussian kernel.

We first incorporated a slow event-related design anchored to the onset of the most emotionally salient portion of each film clip, what we will refer to as the “emotional peak” portion. This peak (assessed in seconds) was determined by the researchers and corroborated by two younger and two older adult naïve raters. An interclass correlation analysis was conducted to assess inter-rater reliability. There was adequate agreement amongst raters for determining the emotional peak (α = 0.79). Final peak ratings were determined by the majority consensus among raters (e.g., 3/5 or 4/5 raters) agreeing on a peaks falling within a range of 1–3 s, whereby the average of that range was used as the peak value (in whole seconds). For this first set of analysis, we used a general linear model incorporating task effects for the negative films in the three viewing conditions (passive, selective attention, reappraisal), along with three linear regressors to account for the three runs, at the single subject level. These models were used to create contrasts between conditions of interest. All contrasts utilized an explicit mask that encompassed all of the PFC, anterior cingulate gyrus, and the amygdala (created using MARINA; Walter et al., 2003) in order to focus our analyses on apriori regions of interest.

We contrasted the combined effects of the two regulation conditions relative to passive viewing (selective attention + reappraisal > passive viewing and passive viewing > selective attention + reappraisal), conducting these contrasts both collapsing across the age groups and also separately for each age group. We also conducted an Age × Condition [regulation (selective attention + reappraisal) vs. passive viewing] ANOVA. These first analyses determined potential age similarities and/or differences in neural recruitment when asked to regulate vs. passively view the clips. These initial analyses also addressed our general prediction that older adults would show diminished activation in response to the regulation conditions relative to younger adults.

Next, we assessed distinct effects of the two regulation conditions for younger and older adults. These analyses specifically addressed whether younger and older adults differed in their activity profile as a function of the specific regulatory strategy used. We first examined activation for each age group in contrasts comparing the two regulatory conditions (selective attention > reappraisal and reappraisal > selective attention). To reveal regions that showed an Age (young vs. old) × Regulation Condition (selective attention vs. reappraisal) interaction, we conducted a separate ANOVA.

Although these prior analyses were based on activity at the peak emotional moment of the film clip, a final analytical approach was used to assess whether age differences in the timing of neural activation might contribute to the observed effects. This analytic approach modeled both the instruction-related activity (modeled as an event starting 4-s post-stimulus onset; see Opitz et al., 2012) and peak-emotion activity (modeled as an event occurring at the most emotionally salient portion of the film clip) for the film clips in the selective attention and reappraisal conditions. An ANOVA was used to reveal regions within the amygdala-PFC mask that showed an Age (young vs. old) × Condition (selective attention vs. reappraisal) × Phase (instruction-onset vs. peak-emotion).

For all analyses, differences in activation are reported for regions consisting of at least 10 voxels, active at *p* < 0.005, unless otherwise specified. This combination of threshold and voxel extent has recently been justified as appropriate in studies equally concerned with Type I and Type II error (see Lieberman and Cunningham, 2009), and in the present study represents a more conservative combination because we limited our search space to the amygdala-PFC mask. AlphaSim (B.D. Ward) revealed a slightly higher voxel extent threshold, of 17 voxels, each active at *p* < 0.005, was required to correct for multiple comparisons across this search space at *p* < 0.05. Notations are provided throughout the results tables to indicate clusters that did not reach this voxel extent. For regions that emerged from a Two or Three-Way interaction in any of our ANOVA analyses, we extracted parameter estimates and plotted the activity within a *post-hoc* region of interest (ROI), defining ROIs within Marsbar (Brett et al., 2002) and plotting the activity using REX (downloaded from http://web.mit.edu/swg/rex/) to reveal the basis for the interaction.

## Results

### Imaging results

#### Effects of passive viewing and emotion regulation separately for younger and older adults at the emotional peak

Again, all analyses focused on negative film clip presentation. We first examined the passive viewing > emotion regulation and emotion regulation > passive viewing contrasts separately for each age group (See Tables 2A,B for results when collapsing across age groups). Older adults showed greater activity in left DLPFC, left VLPFC, and OFC for passive viewing relative to emotion regulation (See Table 2C). Only one cluster within DLPFC was revealed in the emotion regulation > passive viewing contrast for older adults (See Table 2D). For younger adults, the contrast resulting in greater PFC activity was reversed. Only one region of right precentral gyrus was more active in the passive viewing > selective attention contrast (See Table 2E). However, several regions emerged in the emotion regulation > passive viewing contrast for younger adults (See Table 2F). These included left ACC, left VLPFC, bilateral DLPFC, and bilateral OFC.

**Table 2:** **Activation across emotion regulation conditions relative to passive viewing**.

**Brain region**	**BA**	***X***	***Y***	***Z***	***K* extent**	***t*-score**	***p*_uncorrected_**
**A. REGIONS OBSERVED IN THE PASSIVE VIEWING > EMOTION REGULATION CONTRAST (COLLAPSED ACROSS AGE)**							
Left post-central gyrus	2	−42	−20	19	40	3.23	0.001
Left precentral gyrus	6^+^	−40	2	12	11	3.24	0.001
Left superior frontal gyrus (DLPFC)	8^+^	−16	31	37	14	2.89	0.003
	8/9^+^	−16	45	36	14	2.96	0.002
**B. REGIONS OBSERVED IN THE EMOTION REGULATION > PASSIVE VIEWING CONTRAST (COLLAPSED ACROSS AGE)**							
Left superior frontal gyrus^*^	6	−20	−3	50	30	3.75	0.000
Right middle frontal gyrus	6^+^	24	−1	48	12	3.12	0.001
Right precentral gyrus^*^	6	34	2	31	101	3.66	0.000
**C. REGIONS OBSERVED IN THE PASSIVE VIEWING > EMOTION REGULATION CONTRAST FOR OLDER ADULTS**							
Left inferior frontal gyrus (VLPFC)	45	−51	20	10	33	3.22	0.002
Left superior frontal gyrus (DLPFC)	9	−10	50	36	212	4.03	0.000
Left medial frontal gyrus (DLPFC)	9^+^	−8	51	18	12	3.12	0.002
Orbitofrontal cortex	10	0	56	3	224	3.89	0.000
**D. REGIONS OBSERVED IN THE EMOTION REGULATION > PASSIVE VIEWING CONTRAST FOR OLDER ADULTS**							
Middle frontal gyrus (DLPFC)	9	32	15	31	28	3.74	0.000
**E. REGIONS OBSERVED IN THE PASSIVE VIEWING > EMOTION REGULATION CONTRAST FOR YOUNGER ADULTS**							
Right precentral gyrus	4^+^	42	−6	18	11	3.30	0.001
**F. REGIONS OBSERVED IN THE EMOTION REGULATION > PASSIVE VIEWING CONTRAST FOR YOUNGER ADULTS**							
Left anterior cingulate cortex (ACC)	32	−10	21	38	192	4.10	0.000
Left inferior frontal gyrus (VLPFC)	11/47	−34	25	−6	53	3.50	0.001
Left middle frontal gyrus (DLPFC)	46	−44	29	34	44	3.41	0.001
Right middle frontal gyrus (DLPFC)	9/46	42	33	37	252	3.69	0.000
Left orbitofrontal cortex (OFC)	10	−48	48	−9	32	3.48	0.001
Right orbitofrontal cortex (OFC)	10	8	67	10	28	4.19	0.000
**G. REGIONS SHOWING AN AGE × CONDITION INTERACTION (COMBINED EMOTION REGULATION VS. PASSIVE VIEWING)**							
Left middle frontal gyrus (DLPFC)	9/46	−46	31	42	35	*F-score* 11.89	0.001
Right superior frontal gyrus (DLPFC)	9/46^+^	44	35	33	11	9.70	0.002
	9/46	28	52	29	152	15.42	0.000

Stereotaxic coordinates based on the Talairach atlas (Talairach and Tournoux, 1988). All coordinates correspond to clusters that met a voxel extent threshold = 17, p < 0.005.

* Nearby cluster observed within a separate conjunction analysis (reappraisal > passive viewing masked with selective attention > passive viewing).

+ Cluster did not reach 17-voxel extent threshold to correct for multiple comparisons at p < 0.005.

#### Regions showing an Age × Condition (combined regulation vs. passive viewing) interaction at the emotional peak

**Figure 1:**
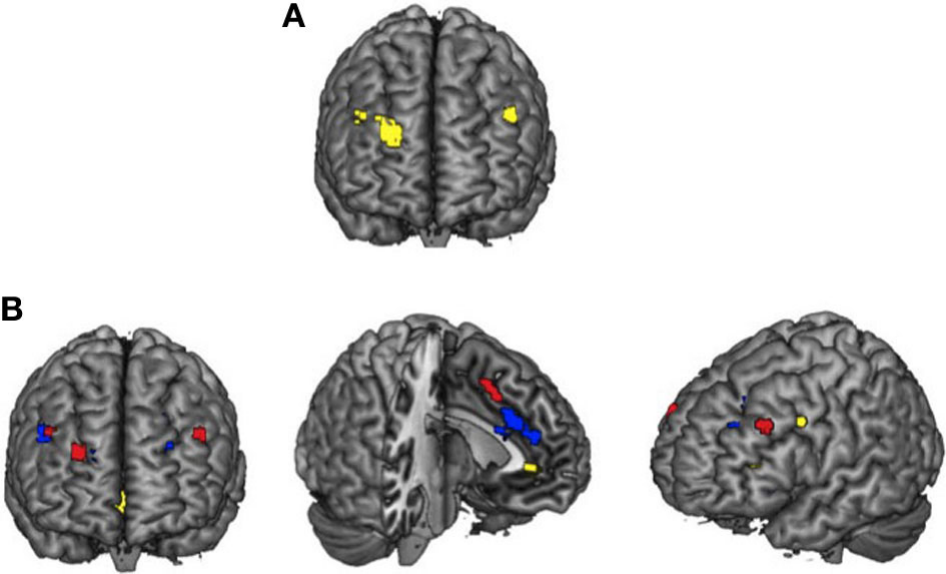
**PFC engagement during emotion regulation.** DLPFC activation observed for the Age × Combined Regulation interaction analyses **(A)** and overlays of activity for selective attention > reappraisal for younger adults in blue, reappraisal > selective attention for older adults in yellow, and the Age × Regulation Condition interaction in red **(B)**.

**A**n ANOVA confirmed a different activity profile for younger and older adults within three regions of DLPFC (Table 2G; Figure 1A). When the parameter estimates from these regions were examined, within two regions of right DLPFC (BA 9/46, peak at Talairach coordinates: 28 52 29; BA 9/46, peak at Talairach coordinates: 44 35 33), older adults showed greater activity than younger adults during passive viewing but less activity than younger adults during emotion regulation. Within the region of left DLPFC (BA 9/46, peak at Talairach coordinates: −46 31 32), younger adults but not older adults showed greater activity for emotion regulation relative to passive viewing^2^.

#### Comparing effects of reappraisal and selective attention for younger and older adults at the emotional peak

The previous analysis suggested greater PFC activity for emotion regulation in younger as compared to older adults. However, this could reflect less overlapping activity within the two regulation conditions for older adults. Thus, we next examined activation patterns between the selective attention and reappraisal condition for younger and older adults. Significant activation patterns occurred for different contrast analyses for each age group: reappraisal > selective attention for older adults and selective attention > reappraisal for younger adults (no significant clusters were observed in the respective opposite contrast for both age groups; Figure 1B). For older adults, activation was greater for reappraisal relative to selective attention in posterior cingulate, ACC, bilateral VLPFC, and OFC (See Table 3A). For younger adults, activity was greater for selective attention relative to reappraisal in bilateral DLPFC, VLPFC, and DMPFC (See Table 3B, Figure 1B).

**Table 3:** **Activation within the reappraisal and selective attention conditions for younger and older adults**.

**Brain region**	**BA**	***X***	***Y***	***Z***	***K* extent**	***t*-score**	***p*_uncorrected_**
**A. REGIONS OBSERVED IN THE REAPPRAISAL > SELECTIVE ATTENTION CONTRAST FOR OLDER ADULTS**							
Left posterior cingulate cortex	31	−16	−33	40	28	4.34	0.000
Left inferior frontal gyrus (VLPFC)	44^+^	−50	9	35	15	3.21	0.002
	45	−38	34	11	55	3.81	0.000
Left orbitofrontal cortex (OFC)	11	−20	40	−12	41	3.92	0.000
Right inferior frontal gyrus (VLPFC)	47	32	42	−12	25	3.60	0.001
Anterior cingulate cortex (ACC)	32	6	43	−2	302	4.35	0.000
**B. REGIONS OBSERVED IN THE SELECTIVE ATTENTION > REAPPRAISAL CONTRAST FOR YOUNGER ADULTS**							
Left middle frontal gyrus (DLPFC)	6	−22	10	47	68	3.57	0.001
	9/46	−24	40	27	25	3.13	0.002
Anterior cingulate cortex (ACC)^*^	32	−10	21	30	282	4.06	0.000
Left inferior frontal gyrus (VLPFC)^*^	47	−36	25	−5	119	3.53	0.001
Right middle frontal gyrus (DLPFC)^*^	9/46	48	29	30	45	3.37	0.001
Left superior frontal gyrus (DMPFC)	8	−12	31	43	62	3.79	0.000
**C. REGIONS SHOWING AN AGE × REGULATION CONDITION INTERACTION (REAPPRAISAL VS. SELECTIVE ATTENTION)**							
Left middle frontal gyrus (DLPFC)	9/46	−44	33	32	19	*F*-score 7.00	0.001
Right middle frontal gyrus (DLPFC)	46^+^	46	31	33	11	6.13	0.003
Anterior cingulate gyrus (ACC)	32	−4	12	45	77	6.47	0.002

Stereotaxic coordinates based on the Talairach atlas (Talairach and Tournoux, 1988). All coordinates correspond to clusters that met a voxel extent threshold = 17, p < 0.005 (no significant clusters revealed in the opposite contrast for each age group).

* Nearby cluster observed within a separate conjunction analysis (selective attention > reappraisal masked with selective attention > passive viewing).

+ Cluster did not reach 17-voxel extent threshold to correct for multiple comparisons at p < 0.005.

#### Regions showing an Age × Regulation Condition interaction

A significant Age *×* Regulation Condition interaction (Table 3C, Figure 1B) was observed within a region of ACC (BA 32; peak at Talairach coordinate: −4 12 45), right DLPFC (BA 46; peak at Talairach coordinate: 46 31 33), and left DLPFC (BA 9/46; peak at Talairach coordinate: −44 33 32).Although these regions were similar to those revealed in the younger adults' contrast of selective attention > reappraisal, the regions were not overlapping (compare red and blue regions in Figure 1B). In each of the regions identified by this ANOVA, younger adults showed similar activation for the two regulation conditions while older adults showed greater activity for reappraisal as compared to selective attention, particularly within the right DLPFC region. Thus, this ANOVA revealed that older adults showed greater differentiation in PFC recruitment for the two regulatory strategies than did younger adults.

#### Phase analysis: activity at film-onset and emotional-peak

**Figure 2:**
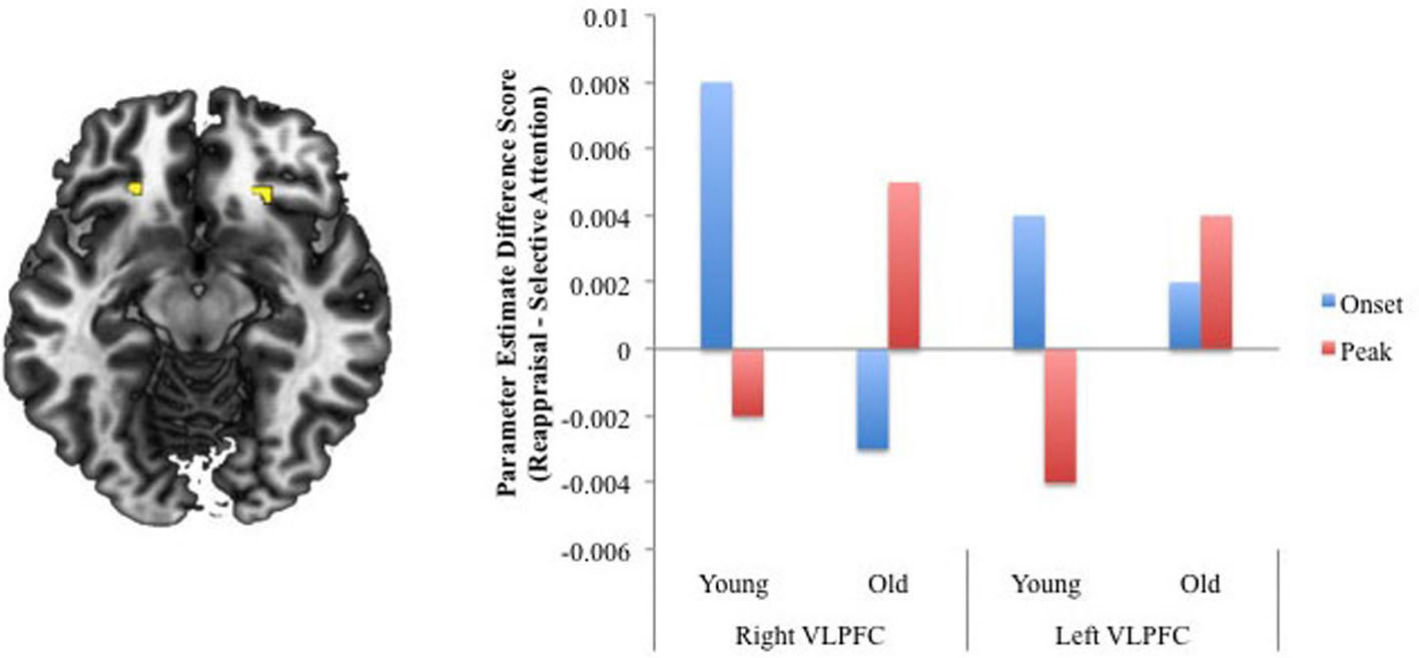
**Regions revealed in the Age × Regulation × Phase interaction analysis.** For ease of presentation, we created difference scores for activity at the onset and emotional peak for the selective attention and regulation conditions separately for younger and older adults. Scores greater than 0 indicate greater reappraisal-related activity and scores less than zero indicate greater selective attention-related activity.

To clarify whether differences noted above might be due to age differences in the timing of regulatory processes, the next analysis examined age differences in activity at instruction-onset and at the emotional peak of the negative videos across the two regulation conditions. An Age × Condition × Phase ANOVA was assessed. Focusing on the three-way interaction, activation was revealed within two regions of VLPFC, although the voxel extent in neither region reached the 10-voxel cutoff (*K* = 5 voxels and 8 voxels); thus, this activity must be interpreted tentatively. Parameter estimates extracted from each VLPFC region(BA 47, peak at Talairach coordinates: 24 25 −13, *p*_uncorrected_ = 0.003, 8 voxels; −22 31 −8, *p*_uncorrected_ = 0.008, 5 voxels) revealed that activity was greater for reappraisal than for selective attention during the onset of the video relative to the emotional peak for younger adults (activity at the peak was greater for selective attention than for reappraisal). For older adults, activity within right VLPFC was greater at the emotional peak during reappraisal than selective attention, while activity was greater at the onset for selective attention than reappraisal. Within left VLPFC, onset and peak activity was slightly greater for reappraisal compared to selective attention for older adults (Figure 2). These results suggest that, particularly within right VLPFC, compared to younger adults, older adults may have delayed engagement of regulatory processes during reappraisal.

## Discussion

The current study investigated the neural mechanisms underlying the hedonic regulation of negative affect in a sample of younger and older adults, examining the effect of regulation strategy (reappraisal vs. selective attention) and the time course of strategy implementation on activity within PFC. We first examined potential overlap across the two age groups in activity that was stronger for emotion regulation relative to passive viewing. These results revealed little overlap in PFC regions supporting regulation relative to passive viewing when collapsing our results across age. More age differences than similarities emerged when we examined the age groups separately. The combined effects of the two regulation strategies relative to passive viewing were more robust for younger adults, with activation observed within lateral and medial PFC. This suggests that younger adults may have more common activation within regions implicated in cognitive emotion regulation networks for both strategies than older adults. Thus, older adults might be showing more differentiation with selective attention and reappraisal.

Indeed, when examining distinct effects for each regulation strategy, further age differences emerged. In some PFC regions, there was a complete age-related reversal in the strategy that utilized the most activity: older adults recruited lateral and medial PFC regions more for reappraisal relative to selective attention while younger adults recruited lateral and medial PFC regions more for selective attention relative to reappraisal. Older adults' greater reliance on lateral and medial PFC activation for reappraisal than selective attention could relate to the heightened cognitive demand that older adults must meet in order to engage these challenging reappraisal strategies. If older adults' implementation of reappraisal processes is less efficient, they may require greater neural engagement than younger adults in order to achieve the same successful regulatory outcome.

Partial support for the interpretation that older adults' reappraisal processes may be engaged less efficiently than younger adults' comes from our phasic analysis of the time course of strategy implementation. This analysis revealed that within two regions of VLPFC, activity was greater for reappraisal than for selective attention during the instruction onset of negative videos for younger adults but not until the emotional-peak of the videos for older adults.

This time course shift might reflect different possibilities for older adults' reappraisal deployment. For one, it is possible that delayed reappraisal-related recruitment in PFC reflects neural decrements related to cognitive slowing (Salthouse, 1995). Such neural decrements would suggest that reappraisal processes require significant effort for older adults to deploy, consistent with behavioral evidence arguing that older adults need sufficient time to recruit cognitive resources for preferentially avoiding negative stimuli (Isaacowitz et al., 2009a). It is plausible that older adults needed more time to select out their preferred reappraisal tactic once they made the initial appraisal (Wager et al., 2008), or they might have had difficulty inhibiting prepotent negative affective responses when attempting to reappraise (Winecoff et al., 2011). Because reappraisal is often most effective when processes are deployed before the emotional response has reached its peak (Goldin et al., 2008), older adults may need to recruit more processes than younger adults because they are attempting to regulate a more intense or mature emotional response. In contrast, by engaging processes earlier, younger adults may successfully curtail the development of a strong emotional response and thereby minimize the cognitive resources required for reappraisal. This latter explanation may also relate to why, for younger adults, selective attention may recruit more activity within PFC regions at the emotional peak than reappraisal: early-acting reappraisal may negate the need for subsequent reappraisal during a film clip, whereas attentional selection of hedonic information at an early time point within a film is unlikely to meaningfully impact the need to select hedonic information at a later time point.

In contrast to a neural-limitation account for the timing effects among older adults, it is possible that the time course of PFC recruitment reflects intentional discretion in terms of strategy deployment. While it is possible that reappraisal is more effective before an emotional response reaches its peak, older adults might be sophisticated enough to wait for the necessary moment when a reappraisal strategy needs to be deployed (i.e., when the most emotionally salient portion of an event/stimulus has emerged). Previous studies assessing reappraisal strategies have not done so over a protracted time course (typically only 2–8 s for stimulus presentation; Winecoff et al., 2011; Opitz et al., 2012) or with more dynamic stimuli (static images as opposed to video stimuli). Thus, older adults might be more motivated to engage in proficient reappraisal strategies with stimuli that are particularly engaging and over a time course suited for full reappraisal deployment. Overall, while these timing effects should be interpreted with caution given the small cluster extents revealed in the analysis, the results are suggestive of the importance of considering age differences not only in the magnitude of recruited PFC processes but also in the time course over which the processes are recruited. Future research should attempt to adjudicate how potential age-related differences in the timing of regulation deployment reflects aspects of ability vs. motivation in the successful use of particular emotion regulation strategies.

In addition to revealing that there are some PFC regions that show age reversals in strategy recruitment, the results also demonstrated that there are PFC regions (namely DLPFC and ACC) in which older adults show more differentiation in the strategy (i.e., reappraisal) that elicits the most activity. Younger adults recruited these to downregulate negative affect by both reappraisal and selective attention. Although prior research has not compared reappraisal and selective attention, this general recruitment in younger adults is consistent with studies that have observed heightened lateral PFC, particularly DLPFC, both when reappraising negative stimuli (Ochsner et al., 2002; Phan et al., 2005; McRae et al., 2010) and when examining attentional deployment strategies (including selective attention; see Silvers et al., 2013, and see Corebetta and Shulman, 2002, for a review of the role of DLPFC in attentional control). Conversely, older adults did not show much in the way of overlapping effects of the two regulation strategies within PFC regions. At first glance, these results seem to be in line with recent work suggesting that older adults do not activate lateral PFC regions to the same extent as younger adults when instructed to regulate negative affective responses (Winecoff et al., 2011; Opitz et al., 2012). However, the lack of PFC recruitment when examining common activation of the two regulation conditions as compared to passive viewing appears instead to relate to discrepant activation patterns across the two strategies when compared to passive viewing for older adults and may suggest that the method of hedonic regulation has a greater impact on the neural processes recruited by older adults than by younger adults.

To our knowledge, only two studies (Winecoff et al., 2011; Opitz et al., 2012) have compared samples of younger and older adults during fMRI investigations of cognitive emotion regulation, and both of these studies employed conditions examining age differences in neural activation when hedonically regulating via reappraisal. Results from these two studies revealed greater lateral and medial PFC activation during reappraisal of negative stimuli in younger as compared to older adults. Conversely, our results suggest that older adults show greater reappraisal related activity within lateral and medial PFC. Although our findings could be consistent with work suggesting that emotion regulation is particularly taxing to cognitive control resources for older adults (see Kryla-Lighthall and Mather, 2009; Urry and Gross, 2010), thus requiring more PFC processes, and more time, for older adults to achieve regulation, the question remains as to why our results seem at odds with those of Opitz et al. and Winecoff et al. One possibility, as mentioned earlier, could be the timing of neural recruitment observed. Whether older adults seem to under-recruit or over-recruit PFC regions during reappraisal may depend on whether activity is measured early in a trial or late in a trial. There are also other design and methodological differences that could lead older adults in our study to implement PFC processes in the service of reappraisal. Most notably, we employed dynamic, emotional film clips, as opposed to static IAPS images. Not only were these films temporally extended, allowing older adults time to implement the regulatory strategies, they might have been particularly engaging. This might have enhanced older adults' ability or motivation to utilize a reappraisal strategy that they report using effectively in their daily lives (see Gross et al., 1997; John and Gross, 2004). Support for this possibility, that older adults successfully use reappraisal when motivated to do so, comes from behavioral evidence revealing that older adults might actually be more successful than younger adults at downregulating affective responses to negative videos (Scheibe and Blanchard-Fields, 2009). It will be interesting for future research to compare older adults' regulatory processes across different time courses and with stimuli that elicit varying motivations for regulation.

### Limitations and future directions

Some limitations should be noted. For one, we did not control the type of reappraisal tactic used by younger and older adults. This provides potential confounds in determining differences in activation patterns across age groups for reappraisal. It is possible that the different age groups were utilizing different strategies (see Shiota and Levenson, 2009, for a discussion), but we have no way of knowing which strategy was most preferred or if more than one strategy was engaged during any specific trial. Whether older adults recruit regulatory control regions within the PFC when utilizing more of a positive reframing rather than a distancing/detached reappraisal tactic needs to be addressed further in the future.

We were also limited by our assessment of regulatory outcomes. We did not examine regulation success on a trial-by-trial basis, which would help determine how well individuals engaged a particular strategy when the efficacy of that strategy was likely to still be in mind. We did assess self-reported affect at the end of each scan run, whereby both younger and older adults showed a modest increase in mood after the regulation runs as compared to passive viewing. However, no effects of age or interactions with age and regulation condition emerged. Furthermore, when we assessed stimulus ratings for the video clips after the fMRI session, there was no effect of regulation condition on the ratings. Although it could be susceptible to demand characteristics, assessing affect (i.e., self-reported mood and stimulus ratings) at the end of each trial might have provided a more accurate portrayal as to how well individuals actually performed the strategy and whether the strategy was effective in eliciting the desired regulatory response/outcome.

Finally, while positive videos were also presented to participants, they were not included in the present paper. This was mainly for logistical reasons and to keep our focus on testing hypotheses relevant to the limited literature in this area that has focused on the regulation of negative affect. However, future analyses will include an assessment of age effects on hedonic regulation of positive stimuli to more fully assess how individuals regulate toward positive affective states (by either increasing positive and/or decreasing negative affect).

In spite of the aforementioned limitations, the present study emphasizes that older adults do recruit lateral and medial PFC regions in response to regulatory instructions. They recruit PFC regions more for reappraisal than for selective attention, perhaps reflecting their need to compensate for less efficient or effective cognitive control processing in order to engage challenging regulation strategies. Consistent with this interpretation that older adults' PFC recruitment during reappraisal may be less efficient than younger adults', the timing of reappraisal-related activity in VLPFC was delayed for older adults compared to younger adults. While older adults implemented VLPFC processes the moment reappraisal instructions were given, older adults did not deploy them until the experienced emotion was likely at its peak. The present findings suggest a need for future research to disentangle age differences in the neural underpinnings involved in executing a variety of cognitive emotion regulation strategies and to examine the implementation of these processes over extended time intervals. This line of research may help to explain which strategies are going to be more or less effective for younger and older adults in achieving regulatory success and enhanced emotional well-being.

### Conflict of interest statement

The authors declare that the research was conducted in the absence of any commercial or financial relationships that could be construed as a potential conflict of interest.

## Footnotes

^1^The instructions were embedded within videos for 14 younger and 13 older adults participants across all three conditions, while reappraisal instructions were embedded within videos for all participants. When assessing results from the remaining 20 younger and 17 older adult participants who did not have visual prompts within the selective attention and passive viewing videos, our main results remained relatively unchanged.

^2^It was possible that results of the Age × Condition interactions (both collapsing across regulation condition and separately for selective attention and reappraisal) were primarily driven by age differences in activation within the passive viewing condition. We examined older adult > younger adult and younger adult > older adult contrasts for just the passive viewing condition. Only one significant cluster emerged in these analyses (DLPFC, BA 45/46; Talairach coordinates: 36 26 24), and was observed in the older adult > younger adult contrast. When we used this result as an exclusive mask and re-ran our interaction analyses, our previous results remained unchanged. This suggests that the observed Age × Condition interactions were not primarily the result of age differences in activation within the passive viewing condition.
